# Gastric perforation secondary to an incarcerated paraesophageal hernia

**DOI:** 10.1186/s40792-019-0653-2

**Published:** 2019-06-10

**Authors:** Shota Fukai, Tadao Kubota, Ken Mizokami

**Affiliations:** Department of Surgery, Tokyo Bay Medical Center, 3-4-32 Todaijima, Urayasu, Chiba, Japan

**Keywords:** Paraesophageal hernia, Gastric perforation, Gastrectomy

## Abstract

**Background:**

Paraesophageal hernias are usually asymptomatic; however, they can cause serious complications such as necrosis or incarceration-induced perforation. Necrosis usually occurs in the incarcerated portion of the hernia. Here, we report the case of a patient with gastric necrosis secondary to an incarcerated paraesophageal hernia in which the necrotic lesion was outside the hernia sac.

**Case presentation:**

A 91-year-old woman presented with severe abdominal pain and vomiting. A physical examination showed hypotension and a diffusely tender and rigid abdomen. Computed tomography showed a paraesophageal hernia, massive ascites, and free air around the stomach. A laparotomy was performed to treat the upper gastrointestinal perforation. The stomach was incarcerated within the paraesophageal hernia sac. After reducing the stomach, we identified a large perforation on the posterior wall of the gastric fundus. Full-thickness necrosis involving part of the stomach necessitated total gastrectomy. She remained physiologically unstable and her condition deteriorated; she died 2 days postoperatively.

**Conclusions:**

A hiatal hernia can be associated with an ischemic gastric perforation outside the hernia sac.

## Background

Paraesophageal hernias are usually asymptomatic; however, incarcerated hernias may cause serious complications [1]. The estimated risk for emergency surgery is 1% in such cases [2]. We report a case of a patient with an incarcerated paraesophageal hernia in which the gastric body and 50% of the fundus were in the hernia sac, whereas the antrum and remaining portion of the fundus were present in the peritoneal space. The portion of the fundus outside the hernia sac showed an ischemic area with transmural necrosis and a macroscopic perforation. A non-strangulated organ (or part of an organ) within an incarcerated hernia concomitant with strangulation and consequent necrosis of the part outside the hernia sac is rare.

## Case presentation

A 91-year-old woman presented with severe abdominal pain and vomiting. She revealed a 12-h history of abdominal pain that had suddenly worsened and was associated with vomiting 1 h prior to presentation. She was otherwise healthy and denied the regular use of any medications.

**Fig. 1:**
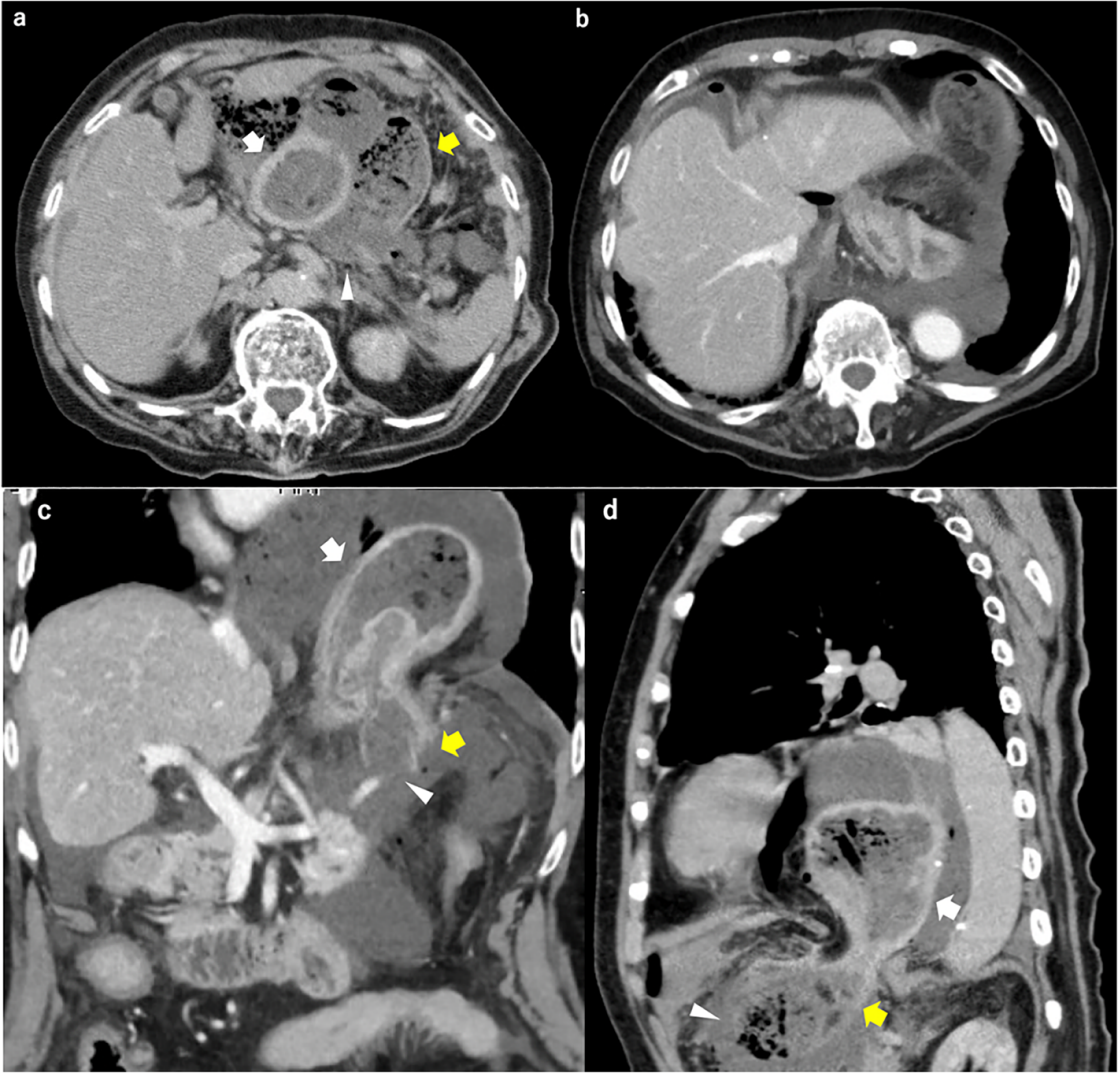
Preoperative computed tomography scan. Preoperative computed tomography scan (**a**, **c**, **d**) showing the gastric fundus and upper body incarcerated within the hiatal hernia (white arrow). The perforation (arrowhead) was located at the fundus outside the sac, which was locally ischemic and necrotic owing to complete cessation of blood supply (yellow arrow). All arteries, including the short gastric artery, were not clear on computed tomography, but arteries flowed into the sac at the hernia orifice (**b**, **c**, **d**) and disappeared

A physical examination showed a blood pressure of 96/58 mmHg, heart rate of 95 beats/min, respiratory rate of 24 breaths/min, and hypothermia. An abdominal examination showed a diffusely rigid abdomen with rebound tenderness over the entire abdomen and reduced bowel sounds. Contrast-enhanced abdominal computed tomography showed a paraesophageal hernia in which part of the stomach was incarcerated (Fig. 1). Free air and ascites were observed in the hernia sac and peritoneal space. The patient developed gastrointestinal perforation-induced peritonitis; however, the association between the incarcerated hernia and the perforation was unclear.

**Fig. 2:**
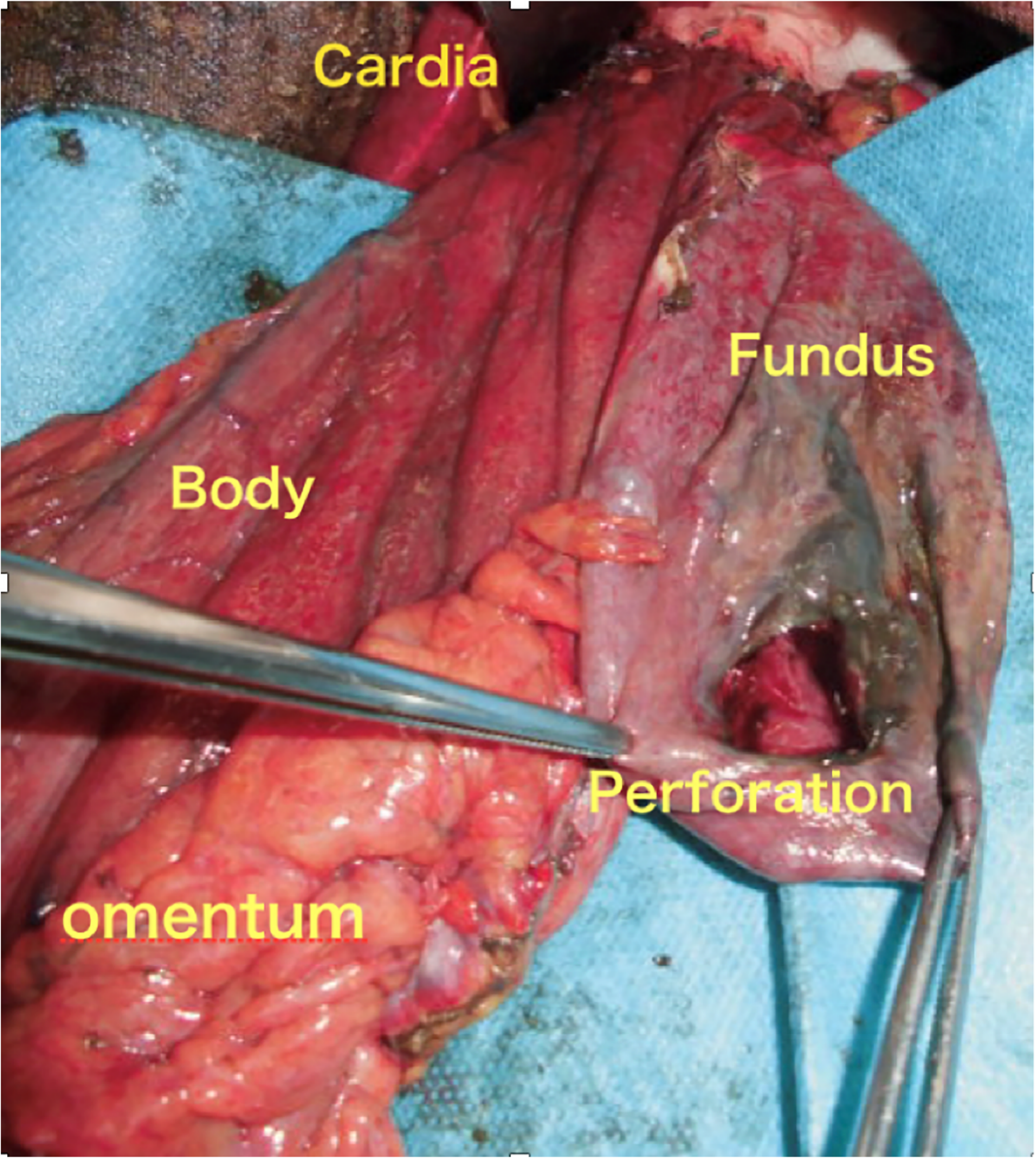
Operative findings and schema. Most of the gastric body and half of the fundus were incarcerated. The antrum and part of the fundus were outside of the hernia sac. After stomach reduction, a 7-cm perforation was seen on the posterior wall of the gastric fundus

**Fig. 3:**
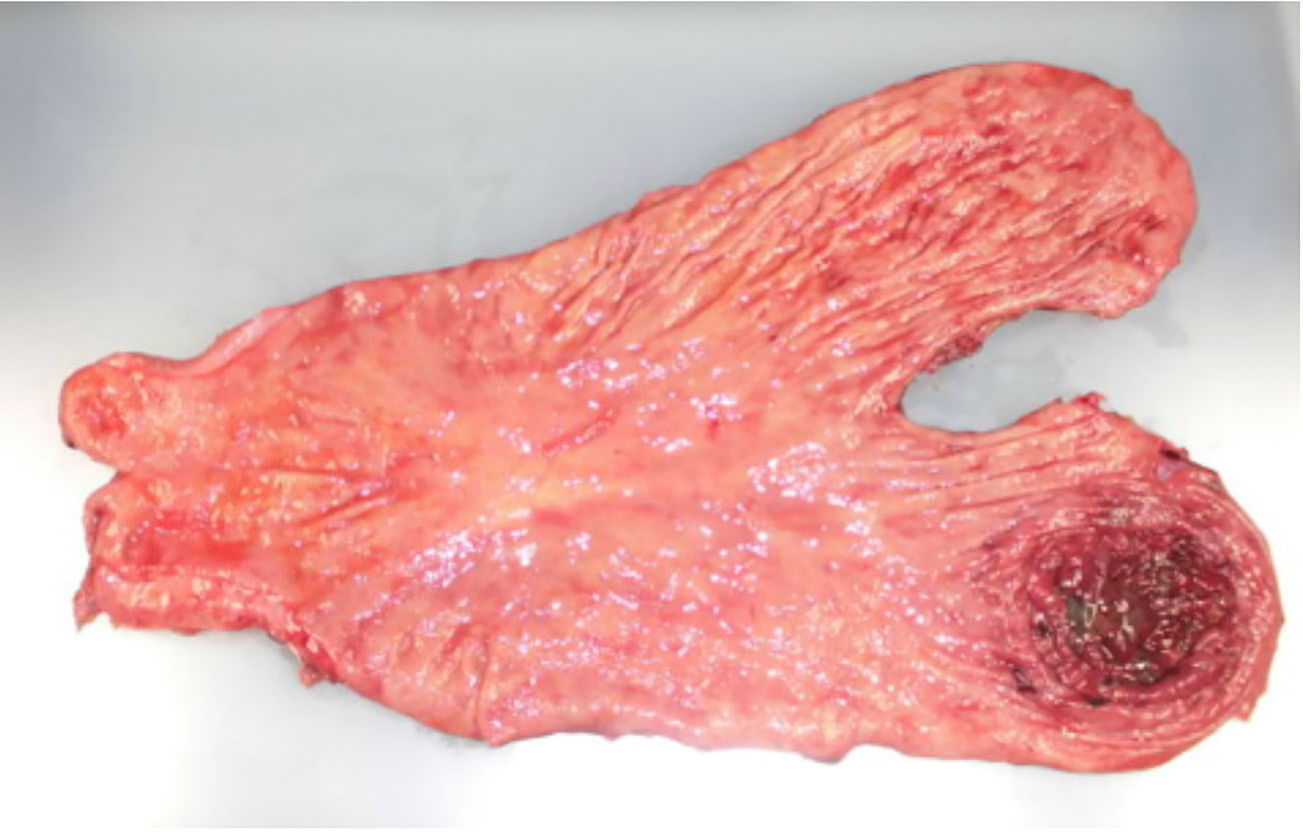
Gross findings of specimen. The perforated area with ischemic necrosis was 7 × 4.5 cm in size at the posterior wall of the gastric fundus, which appeared very thin and dark red

An emergency laparotomy was performed through the upper abdominal midline incision, and we observed that part of the stomach was incarcerated within the paraesophageal hernia sac, which also contained ascitic fluid that appeared like gastric fluid. After reducing the stomach, we detected a perforation measuring 7 cm on the posterior wall of the gastric fundus (Fig. 2). The affected part of the stomach showed full-thickness necrosis, for which we performed total gastrectomy with Roux-en-Y reconstruction. During the operation, the patient’s hemodynamics was unstable; thus, hernia repair only involved closure of the orifice, and fundoplication was not performed. The operative time was 3 h 22 min and the amount of bleeding was limited to 104 ml. The resected specimen showed an ischemic necrosis–induced perforation measuring 7 × 4.5 cm on the posterior wall of the gastric fundus (Fig. 3). Postoperatively, the patient remained hemodynamically unstable despite maximal resuscitative efforts and died on postoperative day 2.

After the operation, the patient remained hemodynamically unstable despite maximal resuscitative efforts. We considered that the distributive shock was affected by the surgery and sepsis on postoperative day 1. However, her condition deteriorated on postoperative day 2. Although we suspected a major leakage, considering her age and hemodynamics, we did not conclude that reoperation would improve her condition effectively. Thus, we informed her family about her condition and decided on nonoperative management. She died on postoperative day 2.

## Conclusions

Four types of esophageal hiatal hernia were identified, and types II, III, and IV are all varieties of paraesophageal hernia [3]. The present case involved type III esophageal hiatal hernia because the phreno-esophageal membrane was stretched and the gastroesophageal junction was displaced above the diaphragm without allowing other organs.

The most notable feature in this case was that, although the perforation site was not within the incarcerated hernia sac, full-thickness necrosis of the gastric wall was evident. The stomach receives its arterial blood supply from multiple sources. Usually, a single artery can maintain adequate organ perfusion, and ischemia is rare. Gastric ischemia is usually associated with complete cessation of arterial blood flow. In this patient, most of the gastric body and 50% of the fundus were incarcerated. The antrum and the remaining portion of the fundus were outside the hernia sac at the time of surgery. Thus, all arteries including the short gastric artery were incarcerated within the sac with complete cessation of blood supply to the fundus outside the hernia sac, which could have precipitated the necrosis. This rare condition could be attributed to the fact that the perforated area was initially incarcerated but underwent spontaneous reduction before surgery. However, this appears to be less likely because there was no evidence on the outer aspect of the stomach to suggest prolonged incarceration of the area. In our opinion, the incarcerated site might not have completely blocked the fundus outside the sac, which was locally ischemic and necrotic owing to complete cessation of blood supply, so the gastric contents in the incarcerated site drained into the abdominal cavity when the fundus outside the sac was perforated and reduced. As the space between the hernia orifice and hernia content expanded owing to the decreasing gastric contents, the fluids and air likely moved into the hernia sac. If our theory is correct, preoperatively judging whether the perforation corresponds to the incarcerated site is difficult.

The optimal operation in this situation remains debatable. We performed a PubMed literature search (2001 to present), and our operation was based on strategies reported in previous studies (Table 1). We identified 3 patients (all elderly women) with perforation associated with an incarcerated paraesophageal hernia [4–6]. The stomach was involved in all patients. One patient showed incarceration at the perforated site. Considering that the incarcerated site could be completely strangulated and necrotic, excision was necessary [6]. Notably, a larger area of incarcerated tissue requires a greater extent of tissue resection.

**Table 1:**
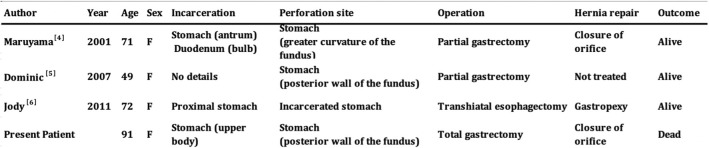
Published reports of ischemic gastrointestinal perforation associated with an incarcerated hiatal hernia

We performed a PubMed literature search (2001 to present), and our operation was based on strategies reported in previous studies. We identified 3 patients with perforation associated with an incarcerated paraesophageal hernia

We identified 2 patients (including our patient) with perforation away from the incarceration site. In these 2 patients, the incarcerated site was not always strangulated and necrotic, and fundal strangulation occurred outside the hernia sac. It would be reasonable to conclude that because the necrotic area was at the site of perforation but outside the hernia sac, only the perforated area required resection. A previous report described favorable outcomes associated with a partial gastrectomy in this context [4]. In our patient, the site of necrosis was identified on the posterior wall of the fundus and the cardia was intact. Retrospectively, a stapled partial gastrectomy may have been reasonable in this case.

Ischemia-induced gastric perforation can occur in association with a hiatal hernia, even in tissue outside the hernia sac.

## Data Availability

The datasets supporting the conclusions of this article are included within the article and its additional files.
